# Modulatory effect of intestinal polyamines and trace amines on the spontaneous phasic contractions of the isolated ileum and colon rings of mice

**DOI:** 10.1080/16546628.2017.1321948

**Published:** 2017-05-26

**Authors:** Manuel Sánchez, Lorena Suárez, María Teresa Andrés, Blanca Henar Flórez, Javier Bordallo, Sabino Riestra, Begoña Cantabrana

**Affiliations:** ^a^ Farmacología, Departamento de Medicina, Universidad de Oviedo, Oviedo, Spain; ^b^ Instituto Universitario de Oncología del Principado de Asturias (IUOPA), Oviedo, Spain; ^c^ Laboratorio de Microbiología Oral, Escuela de Estomatología, Universidad de Oviedo, Oviedo, Spain; ^d^ Servicio de Aparato Digestivo, Unidad de Enfermedad Inflamatoria Intestinal, Hospital Universitario Central de Asturias (HUCA), Oviedo, Spain

**Keywords:** Food composition, polyamines, trace amines, intestinal motility

## Abstract

**Background**: Gastrointestinal motility modulatory factors include substances of the intestinal content, such as polyamines and trace amines (TAs), the focus of this study.

**Methods**: The amines of food, intestinal content and from faecal bacteria of Swiss mice were determined by HPLC and functionally characterised in isolated distal ileum and medial colon rings.

**Results**: Mouse food and intestinal content contain polyamines (spermidine>putrescine>spermine) and TAs (isoamylamine>cadaverine). Intestinal bacteria mainly produce putrescine and cadaverine. The amines inhibited the spontaneous motility of the ileum (0.1-3 mM) and colon rings (0.01-3 mM, with lower IC_50_), with: spermine~isoamylamine~spermidine. Spermine inhibition was tetrodotoxin (TTX)-insensitive, while isoamylamine was TTX-sensitive, suggesting neural control. Mainly in the ileum, isoamylamine (3 mM) elicited acute effects modified by TTX, atropine and propranolol, and suppressed by spermine (3 mM), not being localized at the smooth muscle level. The amines assayed (3 mM), except putrescine and cadaverine in the ileum and isoamylamine in the colon, antagonised acetylcholine (ACh, 0.1 mM)-elicited phasic contractions. Isoamylamine and spermine in colon relaxed KCl (100 mM)-elicited tonic contractions, suggesting an effect on smooth muscle, but did not justify the suppression of motility caused by spermine and isoamylamine.

**Conclusions**: Polyamines and TAs of the intestinal content might act on chemosensors and modulate intestinal peristalsis.

## Introduction

Gastrointestinal motility drives segmentation and peristalsis, and has a complementary role in facilitating the digestion and absorption of nutrients. This consists of well-coordinated spontaneous phasic contractions, occurring in the absence of extrinsic innervation, originating primarily in the pacemaker interstitial cells of Cajal (ICC). They are coupled with smooth muscle and platelet-derived growth factor receptor (PDGFR)-positive cells, forming a functional electrical syncytium, referred to as SIP [1,2]. This electrical syncytium transduces inputs from hormones, paracrine signals, and innervation. Also, it acts in response to chyme via stretch receptors and organic constituents, acting on nutrient sensor [3].

Several biogenic amines, including polycationic molecules, polyamines, and primary amines derived from natural amino acids, are present in the intestinal lumen and incorporated in the chyme. These come from the ingestion of a wide variety of foods [4,5], biliary and pancreatic secretions [6], and are produced by gut microbiota [7]. The dietary polyamines putrescine, spermidine, and spermine are transiently found after meals in the intestinal lumen, being rapidly absorbed by the epithelial cells, where their homeostasis is regulated by biosynthesis, interconversion and release [8,9].

Polyamines that stimulate the growth and healing of the gastrointestinal mucosa [10,11] are also associated with colorectal cancer risk [12] and with the modulation of gastrointestinal smooth muscle motility. Thus, an increase in intracellular polyamines, via the stimulation of ornithine decarboxylase activity, mediates androgen-elicited contractions in mice ileal and colonic smooth muscles [13,14] and in rat heart [15–17]. Extracellular exposure to polyamines relaxed most of the vascular and non-vascular smooth muscles studied [18,19]. This includes the inhibitory effect on guinea-pig gastric smooth muscle [20] and colon [21] motility, although an increase of isometric tension and the facilitation of carbachol-induced contractions in mice ileum has also been reported [14].

The effect of trace amines (TAs) on the gastrointestinal tract is less clear [22]. These have been studied in more depth in olfactory systems as agonists of TA-associated receptors (TAARs) [23] and in the central nervous system (CNS), where they are proposed as neuromodulators or neurotransmitters [24,25]. TAARs are expressed in a variety of peripheral tissues and cells [26], including the gastrointestinal tract of mammals [22,27,28]. Of the TAs, tyramine and β-phenylethylamine contract ileum and vascular smooth muscles, independent of the indirect sympathomimetic effect traditionally assumed, in relation to the activation of TAARs [29,30]. Therefore, amines found in the intestinal lumen could modulate gastrointestinal motility, which might vary depending on the segment of the digestive tract considered and on the type of smooth muscle, longitudinal or circular, whose patterns of activity are related to the physiological function played. These facts led us to identify in mice the amines present in the intestinal content, and those produced by faecal microbiota, and to characterise their effect on the spontaneous phasic contractions in the isolated ileum and colon rings, which mainly involves circular smooth muscles related to the segmentation motor pattern [31,32], critical for the absorption of nutrients and water.

## Materials and methods

### Animals used, intestinal tissue and its content

Male CD-1 Swiss mice 8–10 week old (28–32 g) bred in the facilities of the University of Oviedo (Spain) (Reg. 33044 13A) exposed to a light–dark cycle of 12 h and with free access to water and food (complete feed for Rodents, Global Diet 2914, Harlan Laboratories, Inc.; produced by Mucedola, Milan, Italy) were used. They were sacrificed by decapitation, under anaesthesia, after placing them in an inhalation chamber filled with inhalatory ether (EU Directive 2010/63/EU for animal experiments), following a protocol that was approved by the Institutional Ethics Committee of the Universidad de Oviedo, Spain. The intestine was placed in a Petri dish in Tyrode’s solution (mM composition: NaCl, 137; KCl, 2.7; CaCl_2_, 1.8; MgCl_2_, 1.05; NaH_2_PO_4_, 0.42; NaHCO_3_, 11.9; and glucose, 5.5) at room temperature.

For 12 mice, the content of distal ileum, caecum, and distal colon (faeces) was extracted to determine biogenic amines; for six of them culture anaerobial bacterial media was also inoculated.

### Growth of intestinal bacteria

Intestinal content samples, from ileum, caecum, and distal colon faeces, were collected from the same mice and immediately used to inoculate 2 mL of Anaerobe Basal Broth (Oxoid Ltd., Cheshire, UK). The inoculated medium was cultured for 48 h at 37°C under strict anaerobic conditions (80% N_2_, 10% CO_2_, and 10% H_2_) in an anaerobic chamber (model 1024; Forma Scientific, Marietta, OH). Then, aliquots of 100 µL were used to prepare tenfold serial dilutions. The diluted samples were spread on the surface of non-selective agar media (Anaerobe Basal Agar, Oxoid, UK), and counting of colony-forming units (CFUs) was conducted after 48 h. The rest of the culture was centrifuged (4000*g* for 5 min), and 1 mL of the supernatant was frozen in liquid nitrogen and preserved at −80°C until they were processed to determine the amine content.

The CFUs were counted, at the appropriate dilution, to determine the amount from the different intestinal segments to normalise the amines produced to the media.

Gram stain was used to classify the bacteria as either Gram-positive or Gram-negative.

### Determination of amines in pellet food, intestinal content, and media of cultured intestinal mice bacteria by HPLC

The amines were determined using a pre-column derivatisation method, as previously described [16,33]. Laboratory chow and intestinal content samples were homogenised in 0.5 mL purified water and then treated with sufficient perchloric acid to reach a final concentration of 12% (5 min at 4°C). Equally, 20 µL of anaerobic media, with and without bacterial inoculation, were homogenised in 380 µL purified water and then treated with perchloric acid, to determine the presence of amines. Afterwards, the extracts were centrifuged at 10,000*g* for 30 min, and 0.2 mL supernatants were collected and neutralised with 0.3 mL of a saturated solution of NaHCO_3_. The samples were dansylated overnight (16 h) with 0.5 mL of a solution containing 5 mg mL^−1^ dansyl chloride in acetone. After one extraction with toluene, the toluenic phase was dried under a nitrogen atmosphere, resuspended in 0.2 mL acetonitrile (VWR, Prolabo, France) and then chromatographed in a HPLC (Shimadzu Prominence, Kyoto, Japan) using a C_18_ (2.5 μm, 3.0 × 75 mm^2^) reverse-phase column (XBridge from Waters, Milford, MA, USA) column according to the method described above. The quantification of polyamines was performed using 2-hydroxydiaminopropane as an internal standard. The polyamines were expressed as pmol mg^−1^ of weight sample or pmol mL^−1^ regarding the number of CFUs.

### Isolation of ileum and colon rings of Swiss mice for isometric recordings

The distal segment of the ileum and medial segment of the colon were all cut for the same researcher into rings of 3 mm in width, removing the surrounding connective tissue. The intestinal rings were placed in a 6 mL organ bath containing Tyrode’s solution bubbled continuously with a 95% O_2_ and 5% CO_2_ mixture, to record the spontaneous motility and ACh-elicited contractions. The preparations were also contracted by depolarisation solution, containing (in mM): KCl, 100; NaCl, 40; CaCl_2_, 1.8; MgCl_2_, 1.05; NaH_2_PO4, 0.42; NaHCO_3_, 11.9; and glucose, 5.5. The spontaneous motility and KCl (100 mM)-elicited contractions were recorded by setting the bath temperature at 37°C, or to 35°C, to study ACh-elicited contractions, in order to decrease the spontaneous motility. Isometric responses were measured on a Polygraph 4006 (Letica, Barcelona, Spain) through Pioden UF1 isometric transducers (LCM Systems, Isle of Wight, UK). The tissues were allowed to stabilise for 45 min under a basal tension of 0.5 g before experimentation. During this period, the buffer solution was renewed every 15 min.

### Experimental procedure for motility recordings of ex vivo preparations of ileum and colon rings

After the stabilisation period, the spontaneous motility of the ileum and colon was recorded for 30 min. Afterwards, the preparations were exposed to cumulative concentrations of the corresponding amine assayed: putrescine, spermidine, spermine, isoamylamine, or cadaverine, with an interval of 30 min between consecutive concentrations, up to 3 mM. Then, the drug was washed out by removing and replacing the incubation solution, and measurements were recorded for another 30 min.

The role of intestinal innervation on the effect of spermine and isoamylamine on the ileum and colon was studied by the addition of tetrodotoxin (TTX) (0.6 µM), a selective blocker of voltage-dependent Na^+^ channels [34], to the organ bath 20 min before the amine assayed. The role of β-adrenoceptors on the effect of spermine and isoamylamine on the spontaneous motility of the ileum, and on isoamylamine (3 mM)-elicited acute effects, was pharmacologically studied by adding 0.6 µM of a non-selective β-blocker, propranolol. The functional presence of β-adrenoceptors, in ileum and colon, was characterised by the observation of the response to the addition of isoproterenol (1 µM), a non-selective agonist, to the organ bath. The role of cholinergic receptors was assessed by prior incubation with 1 µM of the antagonist atropine. The influence of spermine (3 mM) on isoamylamine (3 mM)-elicited acute effects was also characterised.

The effect of amines, polyamines, and TAs was also studied on ACh-elicited contractions in the ileum and colon. First, the effect of ACh was established by its addition to the organ bath in a cumulative-concentration manner (0.3 µM–0.3 mM) until the maximum contraction was reached (*Emax* = 100% of response). To study the effect of amines on ACh-elicited contraction, the tissues were contracted by a single concentration of ACh, 0.1 mM. Then, ACh was removed from the organ bath by replacing the incubation media and, with an interval of 30 min after the relaxation of the preparations were reached, the tissues were contracted again with ACh (0.1 mM). The response was reproducible, which allowed the effect of amines to be tested by adding these (at 3 mM) to the organ bath 20 min before a new exposure to ACh (0.1 mM). The effect of lower concentrations (100 µM) of putrescine and spermine on ACh-elicited contractions were also studied at 70 min incubation.

The effect of polyamines and TAs was also studied on the sustained or tonic contraction elicited by 100 mM of KCl in the ileum and colon preparations. This extracellular solution depolarises the tissues to around −11 mV, according to the equilibrium potential for K^+^ obtained from the Nernst equation. The amines were added to the organ bath, at a concentration of 3 mM, when the tonic contractions were stable. Only one drug was assayed in each tissue. In some preparations, the possible interaction between spermine or isoproterenol on isoamylamine-elicited relaxation was studied. Also, the effect of the β-blocker propranolol (0.6 µM) on spermine or isoamylamine elicited relaxation.

The recordings were scanned to be digitalised by means of the software GetData Graph Digitizer (version 2.26.0.20, S. Fedorov).

### Drugs

The following drugs were used: putrescine (tetramethylenediamine), spermidine (*N*-(3-aminopropyl)-1,4-butanediamine), spermine (*N,N*′-bis(3-aminopropyl)-1,4-butanediamine), isoamylamine (isopentylamine: 1-amino-3-methylbutane), cadaverine (cadaverine dihydrochloride), TTX, ACh (acetylcholine chloride), isoproterenol (1-(3ʹ,4ʹ-dihydroxyphenyl)-2-isopropyl-aminoethanol hydrochloride), propranolol (1-(isopropylamino)-3-(1-naphthyloxy)-2-propanolol), and 2-hydroxydiaminopropane were from Sigma. The drugs were dissolved in purified water with a resistance of 10–15 MΩ cm.

### Calculation and statistical analysis

The spontaneous motility, amplitude, and frequency were expressed as absolute values in mg of contraction, with respect to the maintained tone, and contractions per minute. The cumulative concentration effects of the polyamines (putrescine, spermidine, and spermine) and TAs (isoamylamine and cadaverine) modifying the spontaneous motility of ileum and colon rings were expressed as the percentage of the amplitude and frequency of the spontaneous contractions in the absence of the drugs studied. The half inhibitory concentration (IC_50_) of spontaneous motility, in the presence of the amines studied, was calculated by fitting the concentration–response curves with the Hill equation of the form: Inhibitory effect = *Imax*/(1 + (IC_50_/(Drug))*^n^*), where IC_50_ is the concentration that produces 50% of the *Imax* and *n* is the apparent Hill coefficient (Igor Pro V6.3.5.5, WaveMetrics Inc., Oregon, USA).

The transient effect of isoamylamine (3 mM)-elicited raised tone and amplitude of contractions, in the absence or the presence of antagonists (TTX 0.6 µM, isoproterenol 1 µM, propranolol 0.6 µM, atropine 1 µM, or spermine 3 mM), was expressed as the percentage of effect with respect to the amplitude of the spontaneous phasic contractions of the control. The frequency of contractions was expressed as contractions per minute. In addition, the amplitude of the induced transient contractions was normalised dividing them by the values of the maximum contraction in each case. This allows the normalised amplitude of the contraction to be plotted against the frequency of the events to each magnitude of contraction normalised.

The cumulative concentration–response to ACh (0.3 µM–0.3 mM) was expressed as a percentage of the maximal contraction to this agonist (*Emax*), considered 100%. The half-effective concentration (EC_50_) of ACh-elicited contractions were equally calculated by fitting the concentration-response curves with the Hill equation.

The effect of amines on ACh (0.1 mM)-elicited contractions was expressed as the percentage of the contraction elicited in the absence of the amines, and the time constants (τ) of contraction and decay of the exponential fitting in seconds. The tonic phase of contraction was expressed as a percentage of the phasic one.

The magnitude of the spontaneous contractions and the elicited by ACh and KCl, in ileum and colon, were also normalised with respect to mg of tension exerted on the tissues, being expressed in mg of contraction.

The data were expressed as the mean ± standard error of the mean (SE) for a number of at least five different tissues, corresponding to equal numbers of animals in each case. Statistical significance was calculated by means of the Student’s *t* test for unpaired and paired values, considering values of *p *≤ 0.05 as significant. A one-way or two-way between-groups analysis of variance (ANOVA) with the post-hoc test, Tukey’s honest significance test (HSD), was conducted to explore the impact of different amines in the food and intestinal segments, as well as the intestinal bacteria production, and their concentrations on the amplitude and frequency of the spontaneous contractions of isolated preparations of ileum and colon, as well as on the effect of amines studied on ACh (0.1 mM)-elicited contractions.

The analysis of motility was performed with the scientific software Axograph X (version 1.4.4, Berkeley, CA, USA) and Igor Pro, and the statistical calculations by means of IBM SPSS Statistics version 22.0 (IBM Corp.) and Epidat version 3.1 (Conselleria de Sanidade, Xunta de Galicia, Spain, 2006).

## Results

### Content of amines in mice pellet foods, chyme of the distal ileum and caecum, and in rectal faeces of mice and the products generated by the intestinal bacteria of these intestinal segments

The polyamines putrescine, spermidine, and spermine are present in food, being significantly (ANOVA) more concentrated than those analysed in the chyme of the distal ileum and caecum and rectum faeces (*p *< 0.05). The richest polyamine in food was spermidine, followed by putrescine and spermine. In the intestinal content, spermidine was more concentrated in caecum than ileum and faeces (*p *< 0.01), with this polyamine being more important than the others in the three intestinal segments analysed (*p *< 0.001). The concentration of the TA isoamylamine is higher in the intestinal content than in the food (*p *< 0.01), without significant differences between the intestinal segments. Cadaverine concentration was significantly lower in ileum than food (*p *< 0.01) (Table 1).

**Table 1.** Liquid chromatography (HPLC) determination of polyamines and trace amines in pellet food and intestinal content.

Samples	Putrescine		Spermidine		Spermine		Isoamylamine		Cadaverine		Tyramine	
(pmol mg^−1^)	Mean	SE	Mean	SE	Mean	SE	Mean	SE	Mean	SE	Mean	SE
Pellet food	293.85^§^	29.90	779.79^§^	39.96	144.62^§^	12.24	57.37^§^	2.02	26.22^§^	1.26	-	-
Chyme-distal ileum	39.45	5.96	193.27	25.77	27.38	4.12	249.78	46.27	9.20	2.25	-	-
Chyme-caecum	78.82	8.75	497.30^†^	65.33	35.39	13.16	276.09	47.43	16.25	2.53	-	-
Colon faeces	85.69	16.42	351.02	57.70	26.37	5.86	198.52	31.42	18.35	2.76	-	-

Values are means ± SE of at least four determinations, expressed as pmol mg^−1^ of the sample.

(-): below the level of determination.

^§^*p < *0.05 food *vs* intestinal content; ^†^*p < *0.001 Chyme-caecum *vs* ileum and colon (ANOVA).

The anaerobe growth of intestinal bacteria from ileum, caecum, and colon faeces showed that they were Gram-negative. These released amines to the media, being putrescine significantly (*p *< 0.01) more concentrated, in all the intestinal segments inoculated, with respect to the other polyamines and isoamylamine. Putrescine was only significantly higher than cadaverine in the media of anaerobes from colon faeces (*p *< 0.01), with the amount of cadaverine from these bacteria being more important than spermine, spermidine, and tyramine (*p *< 0.01) (Table 2). The anaerobe media lack of these amines.

**Table 2.** Liquid chromatography (HPLC) determination of polyamines and trace amines produced by cultured intestinal bacteria.

Bacteria culture	Putrescine		Spermidine		Spermine		Isoamylamine		Cadaverine		Tyramine	
(pmol CFU (× 105) mL^−1^)	Mean	SE	Mean	SE	Mean	SE	Mean	SE	Mean	SE	Mean	SE
Chyme-distal ileum	87.60^§^	42.88	0.28	0.08	0.14	0.03	4.20	1.25	38.26	18.21	0.72	0.17
Chyme-caecum	78.39^§^	17.39	0.16	0.03	0.13	0.05	3.49	0.72	35.43	7.87	0.47	0.08
Colon faeces	178.18^§^	33.59	0.23	0.05	0.17	0.03	5.93	1.79	83.39^†^	14.42	0.79	0.16

Values are means ± SE of six determinations, expressed as pmol colony forming units (CFUs) (× 10^5^) mL^−^^1^.

^§^*p < *0.01 putrescine *vs* the other amines; ^†^*p *≤* *0.01 putrescine *vs* cadaverine (ANOVA).

### Effect of the studied amines on the spontaneous motility of isolated rings of the ileum and colon

**Figure 1.**
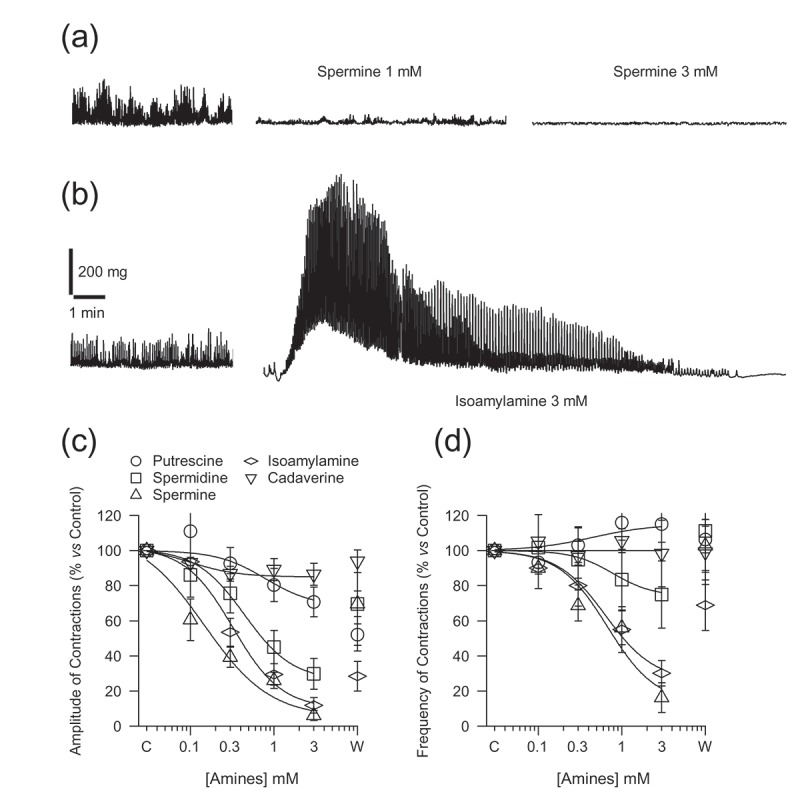
Recording of the effect of spermine (0.3 and 1 mM) (a) and isoamylamine (3 mM) (b) on the spontaneous motility of ileum rings. Concentration–response curves, from 0.1 to 3 mM and the washout (W), of polyamines and trace amines on the amplitude (c) and frequency of the spontaneous contractions (d). The line plot fits the data with a Hill equation with an *n* value close to one. Each point represents the mean ± SE for at least seven mice. The ANOVA showed *p < *0.05 for the effect of putrescine, spermidine, spermine, isoamylamine and cadaverine on the amplitude, and spermidine, spermine and isoamylamine on the frequency of contractions. C: Control, referring the basal motility as 100%.

The isolated preparation of ileum showed spontaneous motility with an amplitude of 165.91 ± 17.88 mg and a frequency of 14.82 ± 0.86 contractions per minute (*n = *143). The addition of the polyamines, putrescine, spermidine, and spermine (Figure 1(a)), and the TAs, isoamylamine (Figure 1(b)) and cadaverine, to the organ bath, at concentrations from 0.1 to 3 mM, decreased the amplitude (Figure 1(c)) and frequency (Figure 1(d)) of the contractions in a concentration-dependent manner. The effect was partially reversible, except for exposure to isoamylamine above 1 mM, in which case only a quarter of the preparations reversed. Isoamylamine at 3 mM initially caused a transient raise of the basal tone, reaching the maximal amplitude of contraction at 54.32 ± 6.99 seconds (*n = *16) of exposure, associated with an increase in the frequency and amplitude of contractions, decreasing afterwards, being followed by a remarkable decrease or suppression of the spontaneous motility (Figure 1(a)).

The efficacy was superior for spermine and isoamylamine than for the rest of amines. The IC_50_ was lower for the amplitude of contractions than for the frequency, which also included to spermidine, putrescine and cadaverine (this was the weakest) (Table 3).

**Table 3.** Values (µM) of inhibitory concentration 50 (IC_50_) of polyamines and trace amines of the amplitude and frequency of the spontaneous contractions of ileum and colon rings.

	Ileum (IC_50_)				Colon (IC_50_)			
	Amplitude		Frequency		Amplitude		Frequency	
Amines	Mean	SE	Mean	SE	Mean	SE	Mean	SE
Putrescine	527.05	174.66	-	-	63.08	5.63	804.83^†^	53.67
Spermidine	520.51	38.18	720.77^†^	85.56	43.31	8.47	259.89^†^	55.56
Spermine	153.2^ϕ,ψ^	10.08	608.62^†^	45.96	25.43^Ϯ^	1.35	68.01^†^	8.24
Isoamylamine	321.23^ϕ^	13.26	1158.3^†^	245.37	32.87	2.86	143.27^†^	17.27
Cadaverine	-	-	-	-	63.2	10.47	82.34	85.04

Values, expressed in µM, were obtained from the Hill equation of the concentration–response curves, for at least seven mice in each case. (-): not determined.

^ϕ^*p *<* *0.001 for ileum amplitude, with respect to spermidine, ^ψ^*p *<* *0.001 spermine *vs* isoamylamine. ^Ϯ^*p *<* *0.05 for colon amplitude, spermine *vs* the rest of amines. ^†^*p < *0.05 frequency *vs* amplitude of ileum and colon.

The ANOVA showed a statistically significant difference in the effect of the concentrations of spermidine (*p *= 0.006) and spermine (*p < *0.001) on the percentage decrease in the amplitude of contractions. However, only spermine produced a concentration-dependent effect on the frequency of spontaneous contractions (*p < *0.001). Regarding TAs, ANOVA showed a concentration-dependent effect of isoamylamine on the amplitude (*p < *0.001) and frequency (*p *= 0.023) of contractions.

Post-hoc comparisons using the Tukey HSD test indicated that the effect of spermine and isoamylamine on the amplitude of the spontaneous motility was different to those of putrescine and cadaverine, at 1 and 3 mM, at a statistically significant level. This was also true for the frequency of contractions with respect to putrescine. At 3 mM, spermidine was also significantly different from putrescine and cadaverine with regard to the amplitude of contractions.

**Figure 2.**
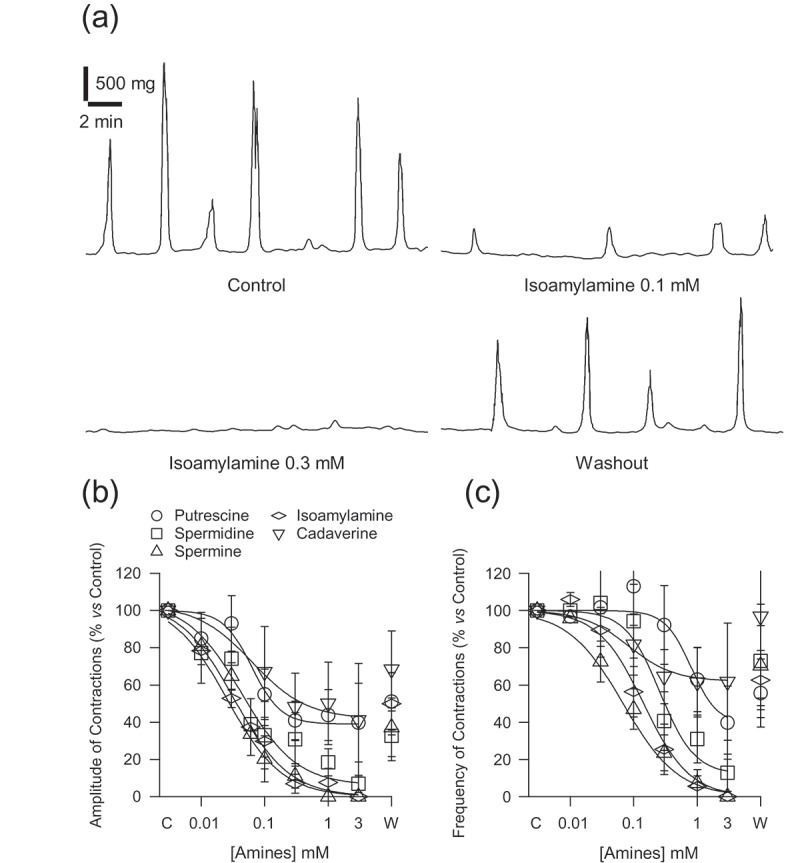
Recording of the inhibitory effect of isoamylamine (0.1 and 0.3 mM) on the spontaneous motility of colon rings (a). Concentration–response curves, from 0.01 to 3 mM and the washout (W), of polyamines and trace amines on the amplitude (b) and frequency of the spontaneous contractions (c). The lines plot the data with a Hill equation with an n value close to one. Each point represents the mean ± SE for at least seven mice. The ANOVA showed *p < *0.05 for the effect of all amines assayed on the amplitude and frequency of contractions. C: Control, referring the basal motility as 100%.

In the colon, the frequency of contractions was significantly (*p < *0.001) slower, 0.43 ± 0.05 contractions per minute, and the amplitude of contractions was larger, 768.87 ± 122.89 mg (*n = *66), than those of ileum. The addition to the organ bath of putrescine, spermidine and spermine, and the TAs, isoamylamine (Figure 2(a)) and cadaverine, from 0.01 to 3 mM, decreased the amplitude (Figure 2(b)) and frequency (Figure 2(c)) of the spontaneous contractions in a concentration-dependent manner. The efficacy was superior for spermine and isoamylamine, showing lower IC_50_ values (Table 3). The effects of these amines were partially reversible after the washout of preparations. Isoamylamine at 3 mM only occasionally produced a transient effect in some preparations, which were qualitatively similar to those described in the ileum.

The ANOVA showed a statistically significant difference in the concentration-effect of spermidine (*p = *0.019), spermine (*p = *0.015) and isoamylamine (*p < *0.001) on the percentage decrease in the amplitude of contractions. The changes in frequency were significant for spermidine (*p = *0.006), spermine (*p = *0.021) and isoamylamine (*p < *0.001). Post-hoc comparisons using the Tukey HSD test indicated that the effect of isoamylamine at 0.3 mM on the amplitude and frequency of the spontaneous contractions was significantly different from putrescine.

### Influence of TTX (0.6 µM), isoproterenol (1 µM), propranolol (0.6 µM), and atropine (1 µM) on the effect of spermine and isoamylamine (3 mM) on the spontaneous motility of isolated intestinal rings

In ileum rings, the incubation with TTX (0.6 µM) increased the basal tone of the tissues, decreasing the amplitude of spontaneous motility, which was the 66.08 ± 7.86% that of the control, without a significant effect on the frequency of contractions, 14.63 ± 1.26 to 12.14 ± 0.88 contractions per minute (*n = *18). In colon rings, TTX (0.6 µM) decreased the amplitude, being 25.83 ± 7.72% of the control, and increased the frequency of contractions, from 2.59 ± 1.38 to 4.95 ± 1.62 contractions per minute (*n = *12).

TTX (0.6 µM) did not antagonise the inhibitory effect of spermine (3 mM) on the amplitude and frequency of spontaneous contractions in ileum. The inhibitory effect of isoamylamine (3 mM) on the amplitude, but not on the frequency of contractions, was partially antagonised, but not significantly, in two of the seven experiments performed, with an average of 34.82 ± 13.37% of the control, being in its absence 11.91 ± 4.4%.

In colon, TTX did not antagonise the inhibition of motility caused by spermine (0.3 mM) or isoamylamine (0.3 mM).

The addition of isoproterenol (1 µM), a non-selective β-adrenoceptor agonist, to the organ bath suppressed the motility of the ileum and colon (data not shown). The effect of the blockade of β-adrenoceptors, by a non-selective antagonist, propranolol, and of muscarinic receptors, by atropine, on the inhibition of intestinal spontaneous motility elicited by spermine and isoamylamine was studied in ileum rings. Propranolol (0.6 µM) decreased the amplitude and frequency of spontaneous contractions, to 83.11 ± 10.59% of the amplitude of the control and 85.35 ± 9.5% of the frequency of contractions. The incubation with propranolol (0.6 µM) did not prevent spermine or isoamylamine (3 mM) inhibition of ileal motility.

Atropine (1 µM) reduced the spontaneous motility of ileum rings to 31.95 ± 15.5% of the amplitude of the control and to 31.68 ± 16.46% of the frequency of contractions. The incubation with atropine (1 µM), did not modify the inhibition of motility caused by spermine (3 mM) or isoamylamine (3 mM).

### Effect of TTX (0.6 µM), propranolol (0.6 µM), atropine (1 µM), and spermine (3 mM) on isoamylamine 3 mM-elicited transient raised tone and the induction of motility of isolated rings of ileum

**Figure 3.**
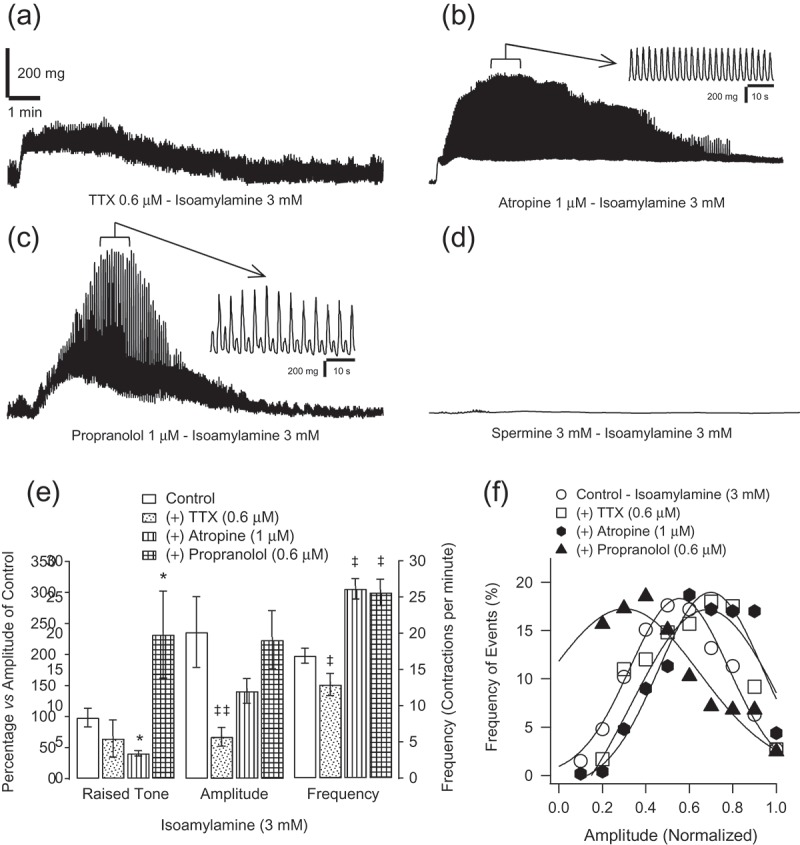
Recordings of isoamylamine (3 mM)-elicited transient effect on ileum rings in the presence of tetrodotoxin (TTX) (0.6 µM) (a), atropine (1 µM) (b), propranolol (0.6 µM) (c) and spermine (3 mM) (d). Average value ± SE (for at least seven different tissues) of the effects of these drugs (except spermine) on isoamylamine (3 mM)-elicited transient increased basal tone, amplitude (expressed as a percentage of the magnitude of the spontaneous phasic contractions in the control rings, in the absence of antagonists) and frequency (in contractions per minute) of contractions of ileum rings (e), *p ≤ 0.05 for unpaired data and ^‡^*p *≤ 0.05 and ^‡‡^*p *≤ 0.01 for paired data, by means of the Student’s *t* test, for at least seven mice, with respect to the control in the absence of amines. Pattern of frequency of the amplitude of the events normalised regarding the maximum amplitude of the induced motility in each preparation (f), with a Gaussian fitting.

Regarding the effect on the acute raised tone in the ileum, previous incubation with TTX (0.6 µM) did not modify isoamylamine (3 mM)-elicited effects, being significantly decreased by atropine (1 µM) and increased by propranolol (0.6 µM), in comparison with the effect in its absence, which was considered the control. Determination of the effects on isoamylamine-induced motility showed that the amplitude of phasic contractions was significantly antagonised in the presence of TTX (0.6 µM) (Figure 3(a–e)). In addition, in the presence of atropine, a latency of 11 seconds elapsed prior to the induction of motility by isoamylamine (Figure 3(b–e)). The frequency of contractions decreased in the presence of TTX (0.6 µM) (Figure 3(e)) and increased when atropine (1 µM) (Figure 3(e)) or propranolol (0.6 µM) (Figure 3(c–e)) was present in the organ bath.

Furthermore, the pattern of motility induced by isoamylamine (3 mM), when the contractions were normalised with respect to the highest amplitude of contraction for each drug, were more homogenous in the presence of TTX (Figure 3(f)) and atropine (Figure 3(b–f)), while a tendency to alternative contractions of large and small amplitude were observed with propranolol (Figure 3(c–f)).

Spermine (3 mM) abolished the transient raised tone and induced motility induced by isoamylamine (3 mM) (Figure 3(d)).

### Effect of amines (3 mM) on ACh (0.1 mM)-elicited contractions in isolated rings of the ileum and colon of mice

ACh (0.3 µM to 0.3 mM) elicited a concentration-dependent contraction of ileum and colon of mice, with EC_50_ values of 42.49 ± 1.87 µM and 21.83 ± 1.01 µM, respectively, under our experimental conditions. At 0.1 mM, ACh elicited a contraction of 70–80% of the maximum in both preparations. The contractions showed a phasic component that was significantly smaller in the ileum than in the colon, of 258.46 ± 46.63 mg (*n = *36) and 564.29 ± 91.78 mg (*n = *36) (*p = *0.0014), respectively, elicited with a time constant (τ) that was significantly slower for the ileum than the colon, 8.93 ± 0.96 s *vs* 6.31 ± 0.44 s (*p = *0.017), respectively. This was followed by a tonic component which was 26.42 ± 3.28% and 18.15 ± 3.33% of the phasic contraction for the ileum and colon, respectively, and with a decay τ that was significantly slower (*p = *0.0019) in the ileum than the colon, 62.23 ± 4.39 s *vs* 43.68 ± 3.72 s.

**Figure 4.**
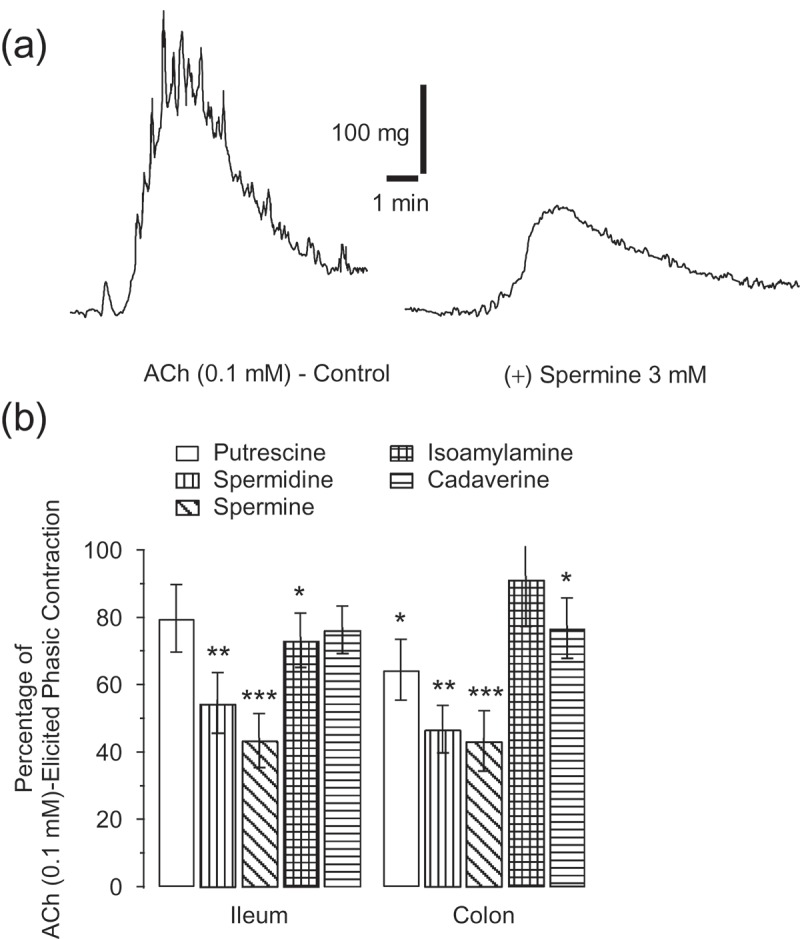
Recording of acetylcholine (ACh) (0.1 mM)-elicited contractions on the ileum and its inhibition by 20 min incubation with spermine (3 mM) (a). Average value ± SE of the percentage of ACh (0.1 mM)-elicited phasic contractions in the presence of polyamines and trace amines assayed (3 mM), in ileum and colon rings (b). *p ≤ 0.05, **p ≤ 0.01, and ***p ≤ 0.001, for paired data by means of the Student’s *t* test, for at least seven mice.

The amines (3 mM) spermidine (*p = *0.003), spermine (*p = *0.001), (Figure 4(a)) and isoamylamine (*p = *0.046) significantly antagonised ACh (0.1 mM)-elicited contractions in ileum rings (Figure 4(b)), and putrescine (*p = *0.039), spermidine (*p = *0.002), spermine (*p = *0.001) (Figure 4(a)), and cadaverine (*p = *0.048), but not isoamylamine, in colon rings (Figure 4(b)). One-way ANOVA showed a statistically significant difference between groups in ileum and colon (*p < *0.001).

Furthermore, in the ileum, spermine significantly increased the τ of contraction which was of 12.65 ± 2.11 s (*p = *0.05). Spermidine and spermine increased the τ of decay of the phasic component, 102.54 ± 16.98 s (*p = *0.05) and 82.99 ± 11.56 s (*p = *0.042), respectively.

In ileum, incubations for 70 min at lower concentration, 100 µM, with spermine inhibited ACh (0.1 mM)-elicited contraction to 86.77 ± 3.83% (*p = *0.019), while putrescine was ineffective.

### Effect of amines (3 mM) on KCL (100 mM)-elicited tonic contractions, influence of isoproterenol (1 µM) and propranolol (0.6 µM) on spermine and isoamylamine effect, and of spermine on isoamylamine, in isolated rings of the ileum and colon of mice

The KCl (100 mM)-elicited phasic contraction was followed by a tonic one in isolated ileum and colon rings. This consisted of an initial transient phasic component, followed by a maintained sustained or tonic contraction. The peak of the phasic component was 630.1 ± 107.38 mg for the ileum (*n = *31) and 1854.43 ± 252.85 mg (*p < *0.001) for the colon (*n = *32). The tonic components were 31.79 ± 4.27% and 28.61 ± 3.09% of the peak of the phasic component for the ileum and colon, respectively.

**Figure 5.**
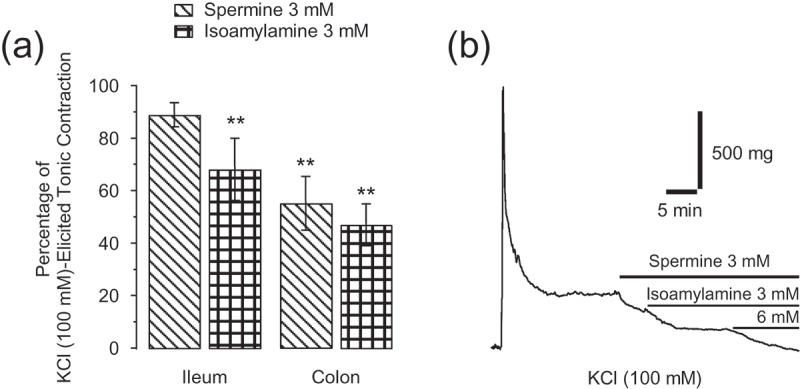
Average value ± SE of the percentage of KCl (100 mM)-elicited tonic contractions in the presence of spermine and isoamylamine (3 mM), in ileum and colon rings (a). **p ≤ 0.01, for unpaired data by means of the Student’s *t* test, for at least seven mice. Recording of KCl (100 mM)-elicited contractions on the ileum and the relaxation of tonic contraction by spermine (3 mM) and isoamylamine (3 and 6 mM) (b).

Isoamylamine (3 mM) significantly relaxed the KCl (100 mM)-elicited tonic component in the ileum and colon rings, with the effect of spermine (3 mM) being weaker in the ileum and similar to that of isoamylamine in the colon (Figure 5(a)). In the presence of spermine (3 mM), isoamylamine (3 and 6 mM) produced an additive relaxation (Figure 5(b)). The rest of the amines did not produce any significant effects.

Isoproterenol (1 µM) relaxed the KCl (100 mM)-elicited tonic component, and in its presence spermine and isoamylamine caused equal relaxation (data not shown). The relaxation to these amines was not reverted by propranolol (0.6 µM) (data not shown).

## Discussion

This study confirms that polyamines and, for the first time shows that, TAs present in the intestinal lumen content may contribute to the modulation of the spontaneous phasic contractions of circular smooth muscle that drive segmentation of the ileum and colon. Differences existed in the efficacy between the compounds assayed, which was superior for spermine and isoamylamine, and on the intestinal segment, mouse colon being more sensitive to a decrease in motility.

The amines in mouse intestinal content showed the highest concentrations in all segments for spermidine, overall the most important in chyme of the caecum, and isoamylamine. Since the presence of biogenic amines depends on the food supplied [35,36], the spermidine source could, at least in part, be from the laboratory chow. The intestinal content could also be substantiated, among other factors, by metabolites produced by the intestinal bacteria, as reported in other species of mice [37]. The intestinal Gram-negative anaerobes produced polyamines, mainly putrescine, and TAs, cadaverine and isoamylamine.

The presence of intestinal amines might be associated with biological effects in the gastrointestinal tract. They were studied with regard to their effects on the epithelial absorption of nutrients [8], and their potential effect in inflammatory bowel diseases [38] and colon cancer adenomas [12]. Polyamines have also been reported to modulate gastrointestinal smooth muscle contractions [13,14,39] and spontaneous motility [20,21,40], based mainly on longitudinal preparations.

Our data show that, as is already known, ileum and colon rings showed spontaneous rhythmic activity *in vitro*, with a higher frequency in the ileum than in the colon, where the characteristic high amplitude and low frequency phasic contractions were present. Exposure to biogenic amines, polyamines, and TAs, present in the intestinal lumen, affected the spontaneous motility in a concentration-dependent manner, producing qualitatively similar effects, without selectivity on the two components of contractions, amplitude and frequency. However, the effect is more pronounced on the amplitude of contractions, being superior for spermine, isoamylamine and spermidine, and colon motility more sensitive than the ileum, with lower values of the IC_50_ values. This and the fact that the amine content is highest in the faeces suggests that an effect on this intestinal segment, is more likely where it may contribute to decrease the motility.

Regarding the inhibitory effects of polyamines and TAs, the lack of a clear dissociation between the effect on amplitude and frequency of the phasic contractions, with the exception of putrescine and cadaverine on the intestine, suggests that they did not specifically target the smooth muscle or pacemaker cells. In a simplistic model, will produce an effect on amplitude without modifications to the frequency of contractions or the opposite, respectively. However, these components are difficult to separate functionally, since changes in the electrical conductance of one of the constituents of SIP-syncytium will affect the activity of the other electrically coupled cells. This syncytium transduces inputs from autonomic nervous system innervation, and other stimuli, increasing the complexity in identifying the cellular target and mechanism of action of the amines.

The role of intrinsic and extrinsic neuronal control on the effects of spermine and isoamylamine on the spontaneous intestinal motility (as the most effective amines) was assessed pharmacologically by establishing the influence of incubation with a blocker of Na^+^-dependent action potentials, TTX [34], and drugs known to bind parasympathetic and sympathetic receptors. As described in circular preparations of the small intestine, but not in the longitudinal ones [41], TTX raised the basal tone of ileum rings maintaining similar spontaneous motility, and only a slight decrease in the amplitude, but not on the frequency of contractions, was observed. This toxin did not modify the basal tone of the colon rings, but decreased the amplitude and increased the frequency of the phasic contractions, as reported in longitudinal preparations [21]. These demonstrated the influence of tonic neural control on spontaneous motility, which might involve the sympathetic and parasympathetic nervous system. This is shown by the alterations in motility due to β-adrenoceptor blockade with propranolol and the suppression of motility in the presence of atropine, a muscarinic antagonist. Muscarinic and β-adrenergic receptors were functionally present, as indicated by the contractions elicited by ACh and the suppression of the spontaneous motility by isoproterenol, a non-selective β-adrenoceptor agonist. These results agree with the modulatory effect of the autonomic nervous system on intestinal intrinsic motility [42–45]. β-Adrenoceptors are widely distributed in the gastrointestinal tract [46–50], including the pacemaker ICC, whose activation inhibits pacemaker currents [51,52], and might produce tonic inhibition of intestinal motility [43].

In our experimental conditions the spasmolytic effect of spermine, in the ileum or colon rings, was produced in the presence of nerve blockade by TTX, ruling out a neural modulation. However, isoamylamine (studied at 3 mM and after 10–15 min of exposure) inhibition of the amplitude of contractions was TTX-sensitive, suggesting a neural effect as reported for other TAs in the CNS [24,25] and the enteric plexus [43].

The effect of amines on the autonomous nervous system were explored, guided by reports that polyamines mediate intestinal effects of autonomous nervous system [14,53,54]. Furthermore, tyramine, a TA, produced an indirect sympathetic effect [53,54] and polyamines bind and activate β_1_-adrenoceptors in rat hearts [55], β_2_-adrenoceptors in the bovine trachea [19], and human stably transfected β_1_- and β_2_-adrenoceptors in CHO cells [56]. However, in intestinal rings neither spermine nor isoamylamine seemed to activate β-adrenoceptors, since propranolol did not modify their inhibitory effects on motility.

The effect of the amines on the intestinal smooth muscle was assessed by ACh- and KCl-elicited contractions. The amines assayed produced a weak anti-muscarinic effect, or interaction with the mechanisms of contraction to ACh, contrary to the potentiation of putrescine and spermine reported on carbachol-elicited contraction in the ileum [14]. The discrepancies might be related to the type of preparation used, longitudinal strips instead of circular ones, and the incubation time of the amines, up to 90 min *vs* 20 min in our study. Although in our experimental conditions, longer incubation times, 70 min, at a lower concentratrion (100 µM) of spermine qualitatively produced the same effect, while putrescine lack of an effect. Our reference for the time of incubation was taken from that necessary to modify the spontaneous motility, whose mechanism we are trying to elucidate. Of the amines assayed, up to 3 mM, only spermine in the colon and isoamylamine in the ileum and colon caused significant relaxation of KCl-elicited sustained contraction, suggesting a weak interference with Ca^2+^ permeability, as in previous reports for the gastrointestinal tract [20,40,57] and other nonvascular smooth muscles [18,19,58]. The mild effect produced indicates that the smooth muscle is not the main target of these amines in inhibiting the spontaneous motility nor was the nervous system involved in this spermine effect. These findings indicate an effect on cells with pacemaker function, while neuronal activation, transduced or not by pacemakers cells, might be involved in the isoamylamine related decrease in amplitude of contractions.

On the other hand, the pharmacological characterisation of the isoamylamine (3 mM)-elicited acute motor response, mainly in ileum, suggests a neural stimulation to increase the frequency and amplitude of contractions (TTX-sensitive), but not the raised basal tone. The autonomous nervous system modulated the effect. Thus, the muscarinic receptors might facilitate the raise of basal tone (decreased by atropine) and limit the frequency of contractions (increased by atropine) in association with a modification in the pattern of amplitude of contractions, which were more homogeneous in the presence of atropine. β-Adrenoceptors might limit the raise of basal tone (facilitated by propranolol) and decrease the frequency of contractions (increased by propranolol). Propranolol also changed the pattern of contractions the most being one third of the maximum. It is interesting that the motor response to isoamylamine was abolished by previous exposure to spermine, but not the relaxation of KCl-elicited tonic contraction, which was additive. The absence of spermine suppression via the smooth muscle or via a neural effect raised the possibility that ICC might play a role in the expression of motor activity induced by isoamylamine.

Overall, the effect of luminal amines seems to be produced via complex interactions involving enteric neurons, the autonomic nervous system, pacemaker cells and/or smooth muscles, which may vary regarding the amines assayed, their possible combinations, and the intestinal segment. The absence of selective antagonists did not allow the possibility of TAAR-mediated response and their cellular location to be characterised. Based on the present results, where isoamylamine and cadaverine functionally modulated intestinal motility, it seems interesting to pursue such a possibility. In relation to this, several TAARs are expressed in the mammalian intestine [22,27,28] and isoamylamine and cadaverine, and some polyamines, might activate TAARs, which have not been described in the intestine [23,59,60]. However, their presence cannot be excluded since the modulation of intestinal motility comprises multiple cell types and it may be difficult to identify whether the receptor expression is below the detection level, if restricted to a minority of cell types.

What seems clear is that the amines present in the intestinal content may act as chemical messengers to locally generate or modulate the pattern of intestinal segmentation, especially spermine and isoamylamine (independent and dependent of nervous system, respectively), which might influence digestive processes. Further investigation should establish the physiological or pathological consequences of alteration in intraluminal content of amines, via the diet or changes in microbiota, as would be the case in antibiotic treatment [61] that may produce functional gastrointestinal disorders. As well as the role of TAAR expression on their effects.
